# Different metabolic features of *Bacteroides fragilis* growing in the presence of glucose and exopolysaccharides of bifidobacteria

**DOI:** 10.3389/fmicb.2015.00825

**Published:** 2015-08-18

**Authors:** David Rios-Covian, Borja Sánchez, Nuria Salazar, Noelia Martínez, Begoña Redruello, Miguel Gueimonde, Clara G. de los Reyes-Gavilán

**Affiliations:** Probiotics and Prebiotics Group, Department of Microbiology and Biochemistry of Dairy Products, Instituto de Productos Lácteos de Asturias – Consejo Superior de Investigaciones Científicas, VillaviciosaAsturias, Spain

**Keywords:** *Bacteroides fragilis*, exopolysaccharides, *Bifidobacterium*, glucose, metabolism, probiotics

## Abstract

*Bacteroides* is among the most abundant microorganism inhabiting the human intestine. They are saccharolytic bacteria able to use dietary or host-derived glycans as energy sources. Some *Bacteroides fragilis* strains contribute to the maturation of the immune system but it is also an opportunistic pathogen. The intestine is the habitat of most *Bifidobacterium* species, some of whose strains are considered probiotics. Bifidobacteria can synthesize exopolysaccharides (EPSs), which are complex carbohydrates that may be available in the intestinal environment. We studied the metabolism of *B. fragilis* when an EPS preparation from bifidobacteria was added to the growth medium compared to its behavior with added glucose. 2D-DIGE coupled with the identification by MALDI-TOF/TOF evidenced proteins that were differentially produced when EPS was added. The results were supported by RT-qPCR gene expression analysis. The intracellular and extracellular pattern of certain amino acids, the redox balance and the α-glucosidase activity were differently affected in EPS with respect to glucose. These results allowed us to hypothesize that three general main events, namely the activation of amino acids catabolism, enhancement of the transketolase reaction from the pentose-phosphate cycle, and activation of the succinate-propionate pathway, promote a shift of bacterial metabolism rendering more reducing power and optimizing the energetic yield in the form of ATP when *Bacteroides* grow with added EPSs. Our results expand the knowledge about the capacity of *B. fragilis* for adapting to complex carbohydrates and amino acids present in the intestinal environment.

## Introduction

The microbes in our body reach levels of up to 100 trillion (10^12^) cells, the majority of which reside in the colon and are anaerobes (Qin et al., 2010). The adult human distal gut microbiota is dominated by two phyla, the Firmicutes and the Bacteroidetes, the genus *Bacteroides* accounting for 20–50% in most individuals (Rigottier-Gois et al., 2003; Mahowald et al., 2009). This group of microorganisms appears during the first few days of life in the intestine of full-term neonates (Arboleya et al., 2015) and remains at low levels (10^7^ cells per gram of intestinal content) in breast-fed infants, increasing after weaning (Mackie et al., 1999). *Bacteroides* species are usually considered as symbionts or mutualists in the human intestine (Hooper and Gordon, 2001). However, in certain circumstances some species can act as opportunistic pathogens (Wexler, 2007). Indeed, the relatively large genome of *Bacteroides* enables these microorganisms to behave both as beneficial and harmful bacteria depending on the host environmental conditions. In this way, the capsular polysaccharide of certain strains of *Bacteroides fragilis* can contribute to the development and maturation of the host immune system (Mazmanian et al., 2005) but is also an important virulence determinant of this bacterium (Wexler, 2007). *Bacteroides* is an anaerobic, bile-resistant, non-spore-forming, and Gram-negative rod. It is a saccharolytic-versatile microorganism able to use dietary or host-derived glycans according to the nutrient availability (Sonnenburg et al., 2005). Members of *Bacteroides* can incorporate amino acids from the external environment (Smith and Macfarlane, 1998). Succinic, acetic, lactic, and propionic acids are produced by *Bacteroides* in variable proportions during fermentation (Rios-Covian et al., 2013). Then, these organic acids and short chain fatty acids (SCFAs) can be utilized by other intestinal microorganisms through cross-feeding mechanisms (Scott et al., 2008) or be partly reabsorbed through the large intestine, thus serving as an energy source for the host (Hooper et al., 2002).

Exopolysaccharides (EPSs) are complex carbohydrate polymers which can be produced by many microorganisms, as is the case of some *Bifidobacterium* strains (Ruas-Madiedo et al., 2007). The intestine is the normal habitat of most species of *Bifidobacterium*, some of whose strains are considered as probiotics and are being included in functional foods (Masco et al., 2005). The synthesis of EPS by bifidobacteria *in vivo* has not yet been demonstrated. However, previous evidence indicates that the presence of bile stimulates the *in vitro* production of EPS by bifidobacteria (Ruas-Madiedo et al., 2006, 2009). In addition, it has been unveiled that in the presence of EPS a relative increase of propionic acid proportions occurs as a result of the metabolic activity of colonic microbiota (Salazar et al., 2008).

The microbiota composition and its functionality in the gastrointestinal ecosystem have been intensively studied, but the dynamics of such microbiota at the metabolic level is not yet well-known. Indeed, in spite of relevant studies on the mechanisms of virulence and pathogenicity of *B. fragilis*, still little is known about the physiology of this microorganism and the adaptation of its metabolism to the gut ecosystem (Wexler, 2007). Although the effective consumption by *B. fragilis* or other intestinal microorganisms of bacterial EPSs has not been unequivocally demonstrated yet, we have recent evidence showing differential growth and metabolic patterns by *B. fragilis* depending on the carbon sources present in the external environment. Thus, a shift toward propionic acid production was found when *B. fragilis* was incubated with bifidobacterial EPS whereas in glucose, acetic acid was the most abundant metabolite formed (Rios-Covian et al., 2013). Therefore, the aim of the present work was to gain insight into *B. fragilis* metabolism grown in the presence of different carbohydrates, including EPSs produced by bifidobacteria, and to examine the results obtained in the light of the role that this bacterium plays as symbiont and opportunistic pathogen in the human gut.

## Materials and Methods

### Bacterial Culture Conditions

Frozen stocks of *B. fragilis* DSM2151 (DSMZ bacterial pure collection, Braunschweig, Germany) were reactivated in Gifu anaerobic medium (GAM) broth (Nissui Pharmaceutical Co., Tokyo, Japan) supplemented with 0.25% (w/v) L-cysteine (Sigma Chemical Co., St. Louis, MO, USA) (named GAMc) and incubated at 37°C for 24 h in an anaerobic cabinet (Mac 1000; Don Whitley Scientific, West Yorkshire, UK) under a 10% H_2_, 10% CO_2_, and 80% N_2_ atmosphere. The pH-free liquid batch cultures of *B. fragilis* were performed in 50 mL of a non-defined peptone and yeast-extract containing basal medium (BM) previously used by us in human fecal cultures (Salazar et al., 2008) and which was subsequently adapted for *Bacteroides* co-cultivation with bifidobacteria (Rios-Covian et al., 2013). The medium had the following composition: peptone (2 g/L), yeast extract (2 g/L), NaCl (100 mg/L), K_2_HPO_4_ (40 mg/L), KH_2_PO_4_ (40 mg/L), MgSO_4_ (10 mg/L), CaCl_2_.H_2_O (10 mg/L), NaHCO_3_ (2 g/L), L-cysteine (2 g/L), bile salts (0.5 g/L), hemin (4 mg/L), Tween 80 (2 mL/L), FeSO_4_ (50 μM), and Na_3_C_3_H_5_O (COO)_3_ (150 μM). A vitamin solution was added, resulting in final concentrations of: vitamin B_12_ (10 mg/L), vitamin K (2 mg/L), vitamin B_1_ (2 mg/L), pyridoxal (1 mg/L), calcium pantothenate (2 mg/L), folic acid (1 mg/L), riboflavin (1 mg/L), biotin (1 mg/L), nicotinic acid (3 mg/L), para-aminobenzoic acid (1 mg/L). The BM was supplemented with 0.3% (w/v) glucose, or bifidobacterial EPS E44 or R1, as specified previously (Rios-Covian et al., 2013). The final pH of the medium ranged between 6.7 and 7.0. A culture of *B. fragilis* incubated in BM without external carbohydrates added was used for comparison in part of the study. The estimate of growth was obtained by measuring the optical density of cultures at 600 nm (OD_600_). Culture media were inoculated with 1% (v/v) of an overnight culture of *B. fragilis* in BM with 1% (w/v) glucose resulting in initial population levels of around 10^6^ CFU/mL.

### EPS Isolation

Exopolysaccharide fractions were obtained from *Bifidobacterium animalis* subsp. *lactis* IPLA R1, a dairy origin strain (Ruas-Madiedo et al., 2006), and from *Bifidobacterium longum* IPLA E44, a fecal isolate from a healthy adult donor (Delgado et al., 2006). EPSs were isolated and purified from the cellular biomass of the producing strains harvested from agar-MRS plates supplemented with 0.25% (w/v) L-cysteine (Sigma Chemical Co.; agar-MRSc) as specified by Salazar et al. (2009b). Briefly, the biomass was collected with ultrapure water and mixed with one volume of 2 M NaOH and gently stirred overnight at room temperature; then, cells were removed by centrifugation and EPSs from supernatants were precipitated with two volumes of absolute cold ethanol for 48 h at 4°C. After centrifugation at 10,000 × *g* for 30 min at 4°C, the EPS fraction was resuspended in ultrapure water, and dialyzed against water for 3 days at 4°C in dialysis tubes of 12- to 14-kDa molecular mass cutoff.

The isolated and purified EPS E44 and R1 fractions contained less than 2.37 and 2.03% (w/w) protein respectively, implying a final protein contribution to the culture medium of about 0.007 and 0.006% (w/v) for EPS fractions E44 and R1, respectively.

### Analysis of SCFA and Organic Acids

Cell-free supernatants from cultures were filtered (0.2 μm). Quantification and identification of SCFA and branched chain fatty acids (BCFAs) was carried out by gas chromatography-mass spectrometry/flame injection detector (MS/FID) using a system composed of a 6890N GC (Agilent Technologies, Inc., Palo Alto, CA, USA) connected with a FID and a MS spectrometry 5973N detector (Agilent Technologies) as described previously (Salazar et al., 2008). Identification and quantification of organic acids was carried out on an HPLC chromatographic system composed of an Alliance 2690 module injector, a PDA 996 photodiode array detector, a 410 differential refractometer detector and Empower software (Waters, Milford, MA, USA). Chromatographic conditions were those indicated previously by Salazar et al. (2009a). Results of SCFA (acetic, propionic), BCFA (isobutyric, isovaleric), and organic acids (lactic, formic, pyruvic, succinic) concentrations were expressed in millimolar (mM). Concentrations of these compounds in media before inoculation (time 0) were subtracted from concentrations in cultures with each carbohydrate added at fixed sampling points during incubation. The sum of acetic, propionic, isobutyric, isovaleric, succinic, formic, pyruvic, and lactic acids was calculated. The molar proportion of each compound was obtained as the concentration percentage with respect to the total SCFA + BCFA + organic acids.

### Amino Acids Analyses

Quantitative determination of amino acids in cell-free supernatants from cultures and in cell-free extracts (CFEs) was carried out by ultra-HPLC (UHPLC) using the method described in Redruello et al. (2013). The chromatographic system consisted of an H-Class Acquity UHPLC coupled to a PDA detector set at 280 nm (Waters). To obtain the CFEs, 10 mL of cultures were centrifuged at 6,500 × *g* for 5 min and concentrated in 1 mL of PBS. Resuspended bacterial pellets were twice broken by sonication for 30 s at 75 W and kept on ice for 1 min between sonication treatments. Cellular rests and unbroken cells were removed by centrifugation at 16,000 × *g* for 10 min at 4°C. CFE were ultra-filtered through a 3-kDa centricon following the manufacturer’s instructions (Millipore, Billerica, MA, USA). One-hundred milliliters of filtered CFE or supernatants were derivatized as described in Redruello et al. (2013) and then one microliter was immediately injected into the chromatographic system. Total protein concentration of the CFE was determined using the Pierce^®^ BCA Protein Assay kit (Thermo Fisher Scientific, Inc., Rockford, IL, USA) according to the manufacturer’s instructions. Results of amino acid concentrations in supernatants are expressed in millimolar (mM), whereas results of amino acid concentrations in CFEs are expressed in mmol/g of total protein. Amino acid levels of the media before inoculation (time 0) were subtracted from those in cultures containing each carbon source.

### Proteomic Analyses

Differences in the proteome of *B. fragilis* growing in the presence of EPS E44 or EPS R1, with respect to glucose, were assessed separately by two-dimensional difference gel electrophoresis (2D-DIGE) as specified by Hidalgo-Cantabrana et al. (2013). In short, CFE from each culture were firstly obtained. Proteins were precipitated by the methanol-chloroform method according to Wessel and Flugge (1984), and resuspended in solubilization buffer (Destreak rehydration solution; GE Healthcare Biosciences, Uppsala, Sweden). Standard 2D gels were conducted first for each condition and were stained with Blue Silver Coomassie (Candiano et al., 2004) in order to obtain reference maps and spot-picking for mass spectrometry analyses. 2D-DIGE was then performed for each EPS and glucose cultures. The manufacturer’s instruction of minimal dye protocol was adopted. Cultures of *B. fragilis* grown with glucose were labeled with Cy3 whereas cultures with each of the EPSs E44 and EPS R1 were labeled with Cy5. Gels were scanned in a Typhoon 9400 scanner (GE Healthcare) at a resolution of 100 μm and analyzed with the 2DImageMaster software (GE Healthcare). *t*-test were run between samples in each gel, and spots displaying statistical differences were excised from Coomassie stained gels and sent to the company Inbiotec (Leon, Spain) for digestion and identification by MALDI-TOF/TOF using standard protocols.

### Gene Expression Analyses

Gene expression was determined by reverse transcription qPCR (RT-qPCR). Ten mL of *B. fragilis* cultures were collected at late exponential phase of growth, cells were mixed with RNA protective bacterial reagent (Qiagen GmbH, Hilden, Germany) and stored at -80°C until use. RNA extraction from cells was performed by phenol/chloroform treatment combined with physical lysis followed by the use of the RNeasy mini kit (Qiagen) as indicated by Ulve et al. (2008). The cDNA was obtained by reverse transcription of 1-μg total RNA using the kit “High Capacity cDNA Reverse Transcription” (Life Technologies, Alcobendas, Madrid). Real-time PCR was performed in an ABI Prism 7500 Fast Real-Time PCR system (Applied Biosystems) and expression levels were calculated by the ΔΔCt method as described previously (Gueimonde et al., 2007). The primers used in this study are listed in Supplementary Table S1, the 16S rRNA gene was used as endogenous control.

### α-Glucosidase Activity

Two mL of culture were concentrated to 1 mL of PBS and CFE was obtained as described in the amino acid analysis section. Total protein determination was as indicated in the same section. The α-glucosidase activity was assayed both in culture supernatants and CFEs by determining the release of *p*-nitrophenol from the substrate *p*-NP α-D glucopyranoside, as described previously (Noriega et al., 2004). Specific activity in both CFEs and supernatants was calculated relative to the amount of cell protein present in 1 mL of culture and was expressed as U/mg.

### Estimation of the Redox Balance

The intracellular redox balance was determined by fluorescence spectroscopy. The fluorescence emissions of buffered cell suspensions at an OD_600_ of 0.6 in 50 mM Tris-HCl buffer pH 7.0 were monitored in an Eclipse fluorescence spectrophotometer (Varian, Inc., Palo Alto, CA, USA). The intensity values corresponding to NAD(P)H were calculated from the 413-nm emission at an excitation wavelength (λex) of 316-nm, whereas for FAD the intensity values were calculated from the 436-nm emission at a λex of 380-nm, as described by Ammor et al. (2004). The redox ratio was deduced from the NAD(P)H- and FAD-related measurements using the equation: redox ratio = FAD intensity/(FAD intensity + NAD(P)H intensity) (Kirkpatrick et al., 2005). Intensity obtained from the suspension buffer was subtracted from those obtained from the samples.

### Statistical Analysis

Statistical analyses were performed using the SPSS-PC software, version 19.0 (SPSS Inc., Chicago, IL, USA). One-way analyses of variance (ANOVA) were run to compare different parameters in culture media added with bifidobacterial EPS (E44 or R1) or glucose or in media without carbohydrates added externally. When appropriate, a *post hoc* least significant difference (LSD) comparison test was applied to determine differences among the different conditions assayed. In the case of the α-glucosidase activity, *t*-tests were run between samples in the different carbohydrates. Experiments were carried out in triplicate.

## Results

### Growth Pattern

Growth pattern of *B. fragilis* varied depending on the culture conditions (**Table 1**). The maximum OD_600_ (OD_600_ max) was significantly higher in cultures with glucose than in the other conditions whereas the lowest values for this parameter were obtained in cultures without carbohydrates added (*P* < 0.05). Considerably lower pH values were obtained in glucose, thus reflecting more acid production and a more active metabolism and growth with this sugar. For most of the experiments performed in this work, we have collected samples when cultures reached their OD_600_ max, which corresponds to late exponential phase (**Table 1**).

**Table 1:** Parameter values of *Bacteroides fragilis* cultures grown in the presence of glucose, EPS E44, EPS R1 or without carbohydrate source added (WCS) at OD_600_ max.

	Glucose	E44	R1	WCS
OD_600_ max	1.16 ± 0.21^c^	0.61 ± 0.06^b^	0.47 ± 0.07^b^	0.23 ± 0.02^a^
Time to reach OD_600_ max (h)	21.71 ± 0.49	23.21 ± 0.28	24.44 ± 0.24	20.6 ± 0.53
pH at OD_600_ max	5.37 ± 0.14^a^	6.92 ± 0.12^b^	6.92 ± 0.04^b^	6.87 ± 0.09^b^

*Different letters indicate significant differences between cultures with different carbohydrates (*P* < 0.05).*

### SCFA and Organic Acids Production Profile

**FIGURE 1:**
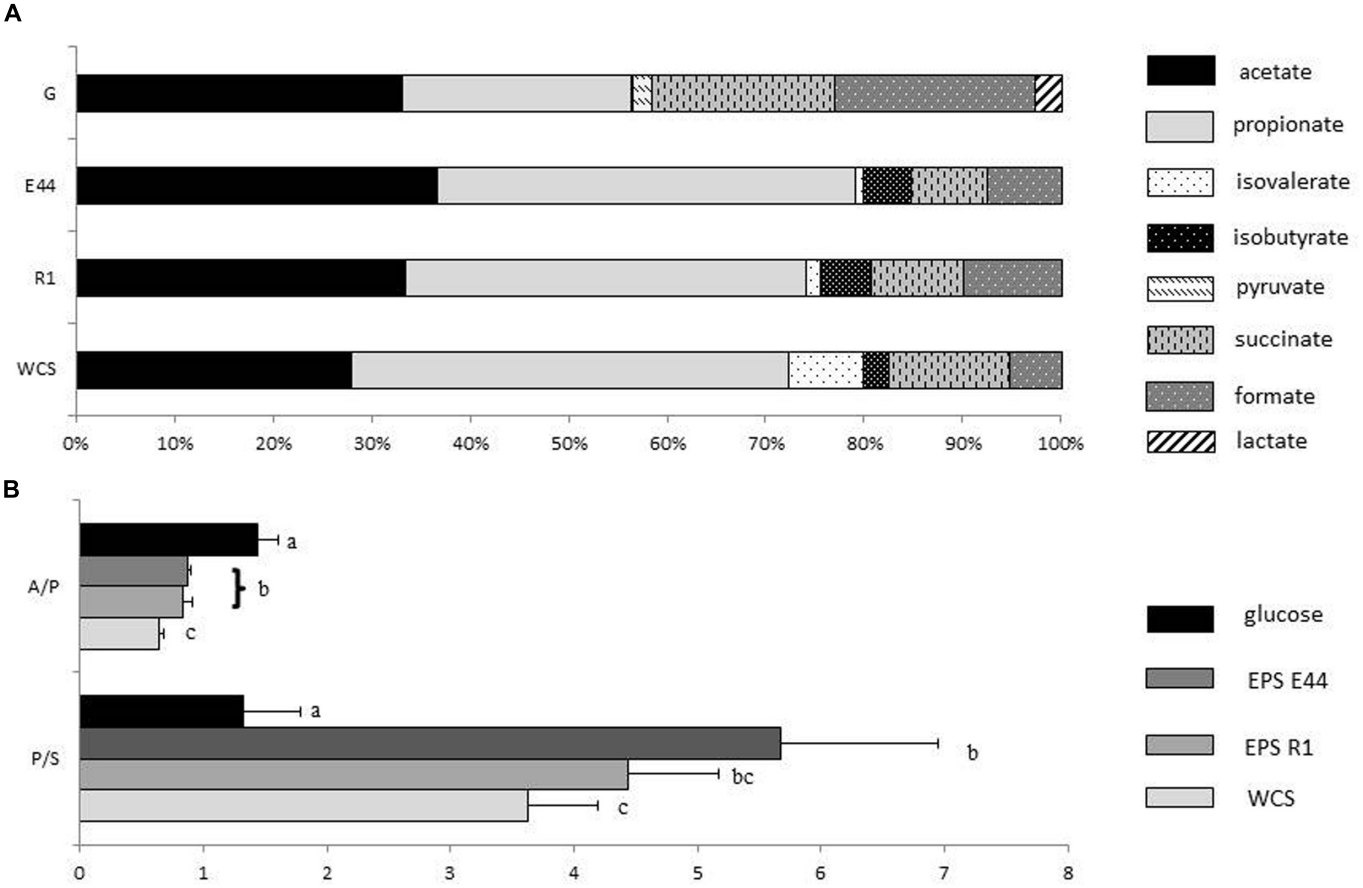
**(A)** Molar proportions of SCFA, and organic acids produced by *Bacteroides fragilis* grown in glucose (G), EPS E44, and EPS R1 and without carbohydrates added (WCS). Acetate 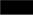; propionate 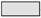; isovalerate 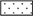; isobutyrate 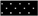; pyruvate 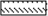; succinate 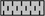; formate 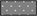; lactate 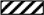. **(B)** Acetate to propionate ratio (A/P) along with propionate to succinate ratio (P/S) of *Bacteroides fragilis* grown in glucose, EPS E44, EPS R1, and WCS. Glucose 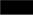, E44 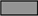, R1 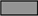, WCS 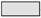. Letters indicate significant differences among cultures with the different carbohydrates (*P* < 0.05). Error bars represent standard deviations. Total metabolite production in glucose: 19.7 ± 1.06, EPS E44: 8.12 ± 1.85, EPS R1: 7.96 ± 0.95, and WCS:4.65 ± 0.39 mM.

Molar proportions of the different SCFA and organic acids produced by *B. fragilis* during growth were clearly different in cultures with additional glucose than in cultures with EPS or without external carbohydrates added (**Figure 1**). Acetic acid was the most abundant SCFA produced in the presence of glucose (33.23%) whereas under other culture conditions the highest molar proportions corresponded to propionic acid (42.31% in cultures with EPS E44, 40.68% in cultures with EPS R1, and 44.23% in cultures without external carbohydrates added). A clear decrease of succinic and formic acids production occurred in cultures with EPS (7.88 and 9.39% for succinic acid and 7.48 and 9.85% for formic acid in EPS E44, and EPS R1, respectively) and without external carbohydrates added (12.19 and 5.22% for succinic and formic acids, respectively) as compared to glucose (18.62% for succinic acid and 20.19% for formic acid, respectively; *P* < 0.05); this happens at the expense of an increase in molar proportions of propionic acid (from 23.13% in glucose to > 40% in the other conditions) and BSCFA (0.22% in glucose in contrast with >5.5% in EPS and without carbohydrates added; *P* < 0.05). Lactic acid appeared in cultures with additional glucose but it was not detected under other conditions. A considerably higher propionic to succinic acid ratio was obtained in cultures with EPS or without carbohydrates (5.68 ± 1.28, 4.43 ± 0.73, and 3.62 ± 0.56 for EPS E44 and R1 and for cultures without carbohydrates added vs. 1.33 ± 046 in glucose; *P* < 0.05). In contrast, *B. fragilis* growing in glucose presented higher acetic to propionic acids ratio (1.44 ± 0.17 in glucose vs. 0.87 ± 0.02 and 0.83 ± 0.08 for EPS E44 and R1, and 0.63 ± 0.04 for cultures without carbohydrates added; *P* < 0.05; **Figure 1**). The production of total SCFA plus organic acids was twice as high in cultures with glucose (19.7 ± 1.06 mM) than in cultures with EPS (8.12 ± 1.85 and 7.96 ± 0.95 mM for EPS E44 and R1, respectively) and was around twofold higher in the presence of these polymers than in media without the addition of carbohydrates (4.65 ± 0.39 mM).

### α-Glucosidase Activity

**FIGURE 2:**
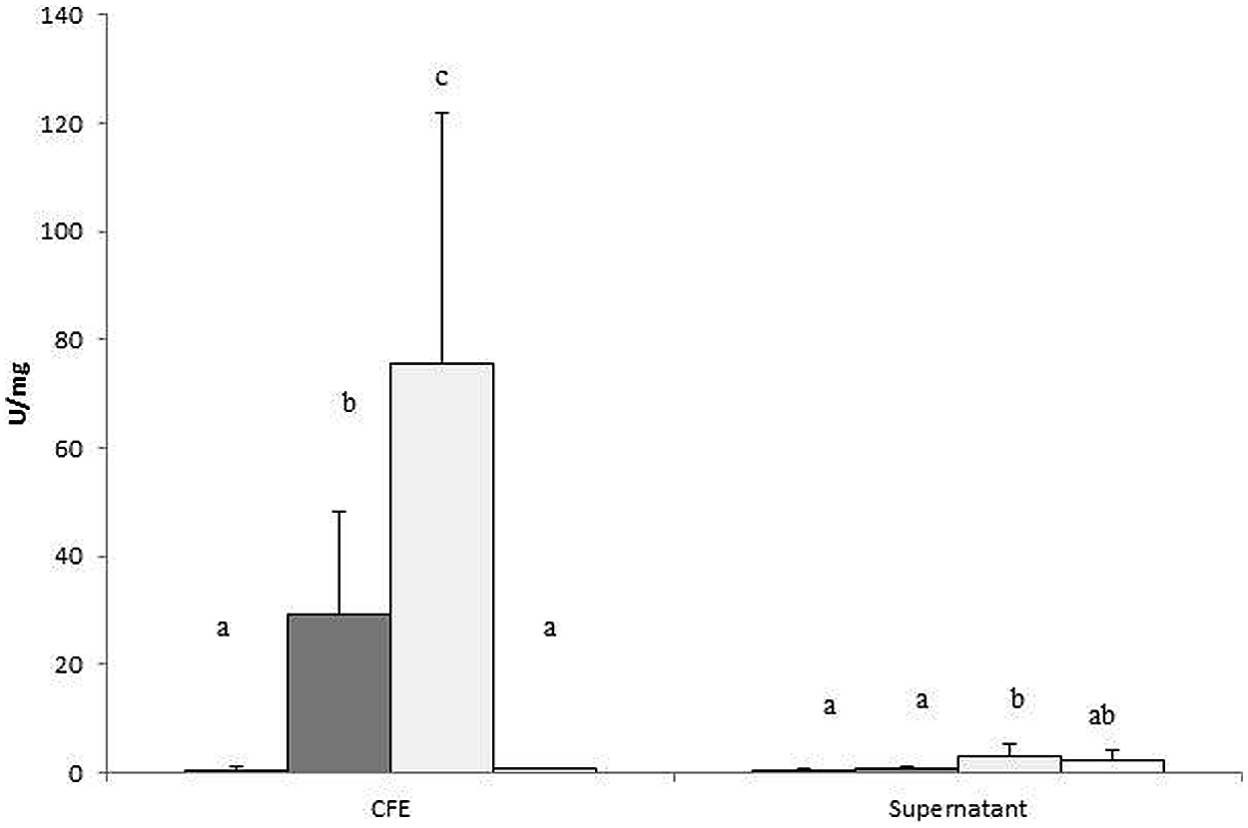
**α-glucosidase activity was determined in cell-free extract (CFE) and supernatants of *B. fragilis* grown in glucose (black bars), EPS E44 (dark gray bars), EPS R1 (light gray bars) and without carbon source added (WCS; white bars), and was referred to the amount of protein present in 1 mL of cell culture and expressed as U/mg protein.** Different letters indicate significant differences among cultures with the different carbohydrates (*P* < 0.05). Error bars represent standard deviation.

Cell-free extract of *B. fragilis* incubated in the presence of EPS E44 and R1 displayed higher α-glucosidase activity than in the presence of glucose, where the amount of this enzymatic activity was negligible (*P* < 0.05; **Figure 2**). No remarkable α-glucosidase activity was found in the supernatants of cultures, neither in supernatants nor CFE of cultures incubated in the absence of carbohydrates added. This indicated that the α-glucosidase activity was mainly intracellular and its production was dependent on the presence of EPS in the culture medium.

### Amino Acids and Ammonia Profiles

**FIGURE 3:**
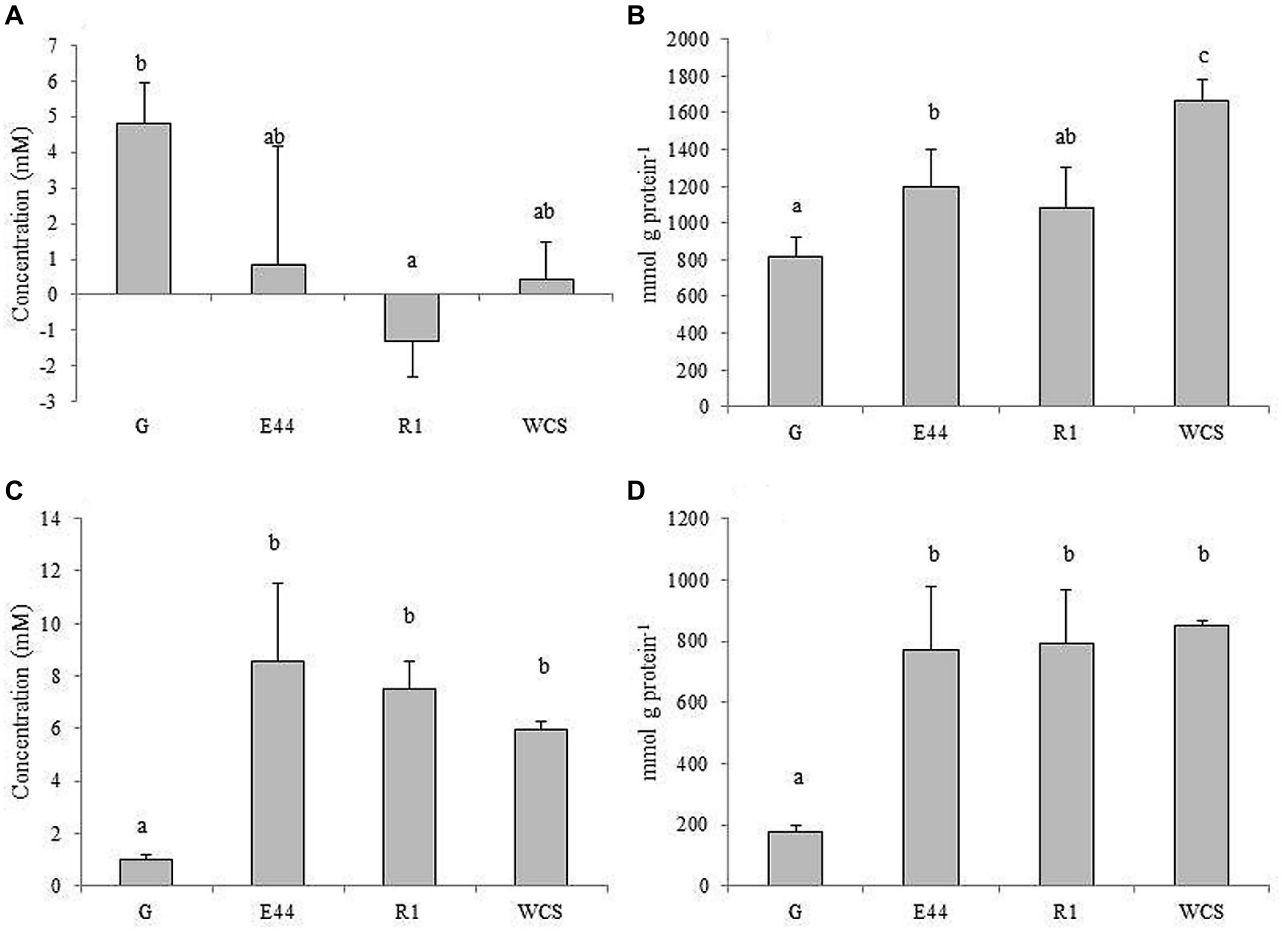
**Total amino acid concentrations in culture supernatants **(A)**, and CFEs **(B)** along with ammonia levels in supernatants **(C)** and CFEs **(D)** of *B. fragilis* DSMZ2151 grown in glucose (G), EPS E44, EPS R1 and in medium without carbohydrate source (WCS).** Different letters indicate significant differences among cultures with the different carbohydrates (*P* < 0.05). Error bars represent standard deviation.

In culture supernatants of *B. fragilis* grown in the presence of EPS or in medium without additional carbohydrates, the levels of total free amino acids hardly increased, or even decreased after incubation, whereas with glucose the concentration of total amino acids clearly rose for the same conditions (*P* < 0.05; **Figure 3A**). In CFE, although levels of free amino acids per mg of cellular protein augmented in all cases, the most pronounced increases occurred in the absence of exogenous carbohydrates. Cultures in the presence of additional EPS displayed intermediate concentrations with respect to the other two conditions (**Figure 3B**). Levels of ammonia increased more in supernatants of cultures with EPS or without carbohydrates added than in cultures with glucose (*P* < 0.05; **Figure 3C**). Intracellular content of ammonia followed the same trend as the supernatants, with a higher accumulation in CFE of *B. fragilis* grown in the absence of carbohydrates and in media amended with EPS, than with glucose (*P* < 0.05; **Figure 3D**). All these factors taken together indicated that, proportionally, more amino acids are taken up by cells, and a concomitant release of ammonia took place when *B. fragilis* was grown in medium supplemented with EPS or in the absence of carbohydrates, as compared with glucose. Intracellular accumulation of amino acids seems to occur to a higher extent in media with EPS or without carbohydrates than in the medium containing glucose (*P* < 0.05).

In order to better understand these phenomena, the profile of amino acid concentrations was analyzed by UHPLC (Supplementary Table S2). Regardless of the culture conditions, specific amino acids were removed to variable extents, from the external medium, whereas others seemed to accumulate. The intracellular level of the different amino acids also differed. Notably, the carbohydrates present in the culture medium affected the profile of some amino acids differently. Thus, glutamic acid, glycine, threonine, GABA, tyrosine, and ornithine accumulated in the supernatants of cultures in the presence of glucose, whereas they were removed or accumulated at significantly lower levels in the presence of one or the two EPSs or in the absence of additional carbohydrates (*P* < 0.05). Conversely, with EPS or without exogenous carbohydrates, lysine and histidine, along with other amino acids, were found in supernatants at significantly higher concentrations than in cultures with glucose (*P* < 0.05). In addition, the intracellular content of six amino acids (aspartic acid, glutamic acid, tyrosine, valine, isoleucine, and lysine) appeared significantly augmented in the absence of carbohydrates added compared to cultures supplemented with glucose or EPS (*P* < 0.05). This indicated a shift in the amino acid metabolism by *B. fragilis* influenced by the type of carbohydrates available in the culture medium.

Further experimentation focused on *B. fragilis* growing in media in the presence of EPS or glucose as we aimed to ascertain the metabolic differences under these two conditions.

### Proteomic Analysis of *B. fragilis* Growing in the Presence of Different Carbohydrates

The protein pool of *B. fragilis* was analyzed by means of 2D-DIGE and the proteome of the microorganism growing in the presence of each of the two EPS was compared with the proteome of the microorganism in glucose (**Table 2**). We focused only on spots corresponding to proteins displaying statistical differences (*P* < 0.05) and production ratios in EPS greater or lower than twofold compared to glucose alone, in at least one of the two polymers. According to this criterion, the production of 10 proteins was found downregulated in cultures with one or both EPSs whereas 13 proteins were upregulated. Cultures of *B. fragilis* with additional EPS R1 presented more variation in the protein-production levels with respect to glucose than cultures with EPS E44. Thus, three proteins were underproduced in the presence of the EPS R1, but not with EPS E44, whereas a unique protein was downregulated with EPS E44 but not with EPS R1; four proteins were found upregulated only in the presence of EPS R1, but not with EPS E44 whilst the contrary was never observed. The presence of some protein spots with the same GI number and molecular mass, but differing in their experimental isoelectric points, suggested the presence of isoforms. Proteins and enzymes whose production was affected by the carbohydrates available in the culture medium belong to several functional COG categories (**Table 2**).

**Table 2:** Identification of *B. fragilis* DSMZ 2151 proteins affected by carbohydrate sources.

COG orthology	Spot no.^a^	Putative function^b^	GI number^c^	Mass^d^	pI^d^	MASCOT score	No. of peptides matched^e^	Cov.	Change fold^f^	
									E44	R1
Carbohydrate transport and metabolism	78	Transketolase	gi| 265763039	72.4	5.5	594	26	52	ND	2.48
	79	Transketolase	gi| 265763039	72.4	5.5	401	21	42	2.32	4.04
	81	Alpha-glucosidase	gi| 265762646	82.1	5.7	743	30	54	7.08	2.97
	87	Pyruvate phosphate dikinase	gi| 53713829	100.6	5.4	564	31	39	3.46	2.37
	89	Pyruvate phosphate dikinase	gi| 53713829	100.6	5.4	410	37	39	3.19	4.39
	90	Pyruvate phosphate dikinase	gi| 53713829	100.6	5.4	629	36	52	3.04	3.86
	105	Pyruvate phosphate dikinase	gi| 60682047	100.6	5.5	840	35	49	3.26	2.67
	101	Galactokinase	gi| 53712943	43.5	5.2	728	22	52	ND	2.29
Lipid transport and metabolism	82	Methylmalonyl-CoA mutase, large subunit	gi| 53715084	79.2	5.6	458	23	43	4.56	2.51
Amino acid transport and metabolism	3	Oxidoreductase (diaminopimelate dehydrogenase)	gi| 60682941	32.5	6.2	938	25	90	-2.06	-2.84
	9	NAD(P)H-utilizing glutamate dehydrogenase	gi| 60682871	49.1	6.1	526	19	53	-6.2	-7.37
	10	NAD(P)H-utilizing glutamate dehydrogenase	gi| 60682871	49.1	6.1	461	16	42	-6.17	-7.60
	11	NAD(P)H-utilizing glutamate dehydrogenase	gi| 60682871	49.1	6.1	430	17	44	-5.50	-7.19
	28	Ketol-acid reductoisomerase	gi| 53715042	38.2	5.2	1100	14	58	ND	-2.48
	62	Acetolactate synthase, large subunit, biosynthetic type	gi| 392698324	61.7	5.1	209	12	25	-2.45	-2.15
Post-translational modification, protein turnover, chaperones	12	Serine protease	gi| 53714036	54.7	6.5	296	14	30	-2.12	-2.30
Translation, ribosomal structure and biogenesis	36	Translation elongation factor 1A (EF-1A/EF-Tu)	gi| 319900910	43.7	5.3	260	8	23	ND	-4.76
	37	Elongation factor Tu	gi| 53715484	43.8	5.2	354	11	32	-2.96	-6.87
	83	Elongation factor G	gi| 265766981	80.4	5.7	489	24	38	2.59	2.83
	84	Elongation factor G	gi| 53715151	80.4	5.6	775	37	51	2.55	2.82
	103	Elongation factor G	gi| 53715468	77.9	5.1	712	23	44	ND	-2.87
	95	50S ribosomal protein L14	gi| 301161079	13.2	9.9	160	6	58	-	-2.03
	99	Translation initiation inhibitor	gi| 53714265	13.1	5.0	88	3	25	-	3.60
Cell wall, membrane, envelope biogenesis	110	Major outer membrane protein OmpA	gi| 53715321	43.3	8.7	361	15	37	ND	4.77
Inorganic ion transport and metabolism	108	Ferritin	gi| 53714336	18.1	5.0	692	12	71	2.35	2.00
Function unknown	39	Hypothetical protein BF2494	gi| 60681974	45.8	5.2	545	18	43	2.30	3.28
	41	Hypothetical protein BF2494	gi| 60681974	45.8	5.2	590	18	42	2.87	4.04
	43	Hypothetical protein BF2494	gi| 60681974	45.8	5.2	574	18	40	2.90	3.75
	92	Hypothetical protein BSHG_4061	gi| 383118833	16.3	5.1	163	8	60	-3.74	ND
	107	Hypothetical protein BF2537	gi| 53713828	20.1	6.3	533	13	79	ND	2.24

*^a^Spot numbers refer to the proteins labeled in 2D-DIGE gels.*

*^b^Putative functions were assigned from the NCBI gene database.*

*^c^GI number in the NCBI database for *B. fragilis* DSMZ 2151.*

*^d^As given by the NCBInr database for *B. fragilis* DSMZ2151. Molecular masses are expressed in kilodaltons.*

*^e^Number of tryptic peptides observed contributing to the percentage of amino acid coverage.*

*^f^Normalized change fold for each protein production derived from cells grown in EPS E44 or R1 with respect to the protein derived from cells grown in glucose. ND, ratio below 2; -, spot not detected.*

**FIGURE 4:**
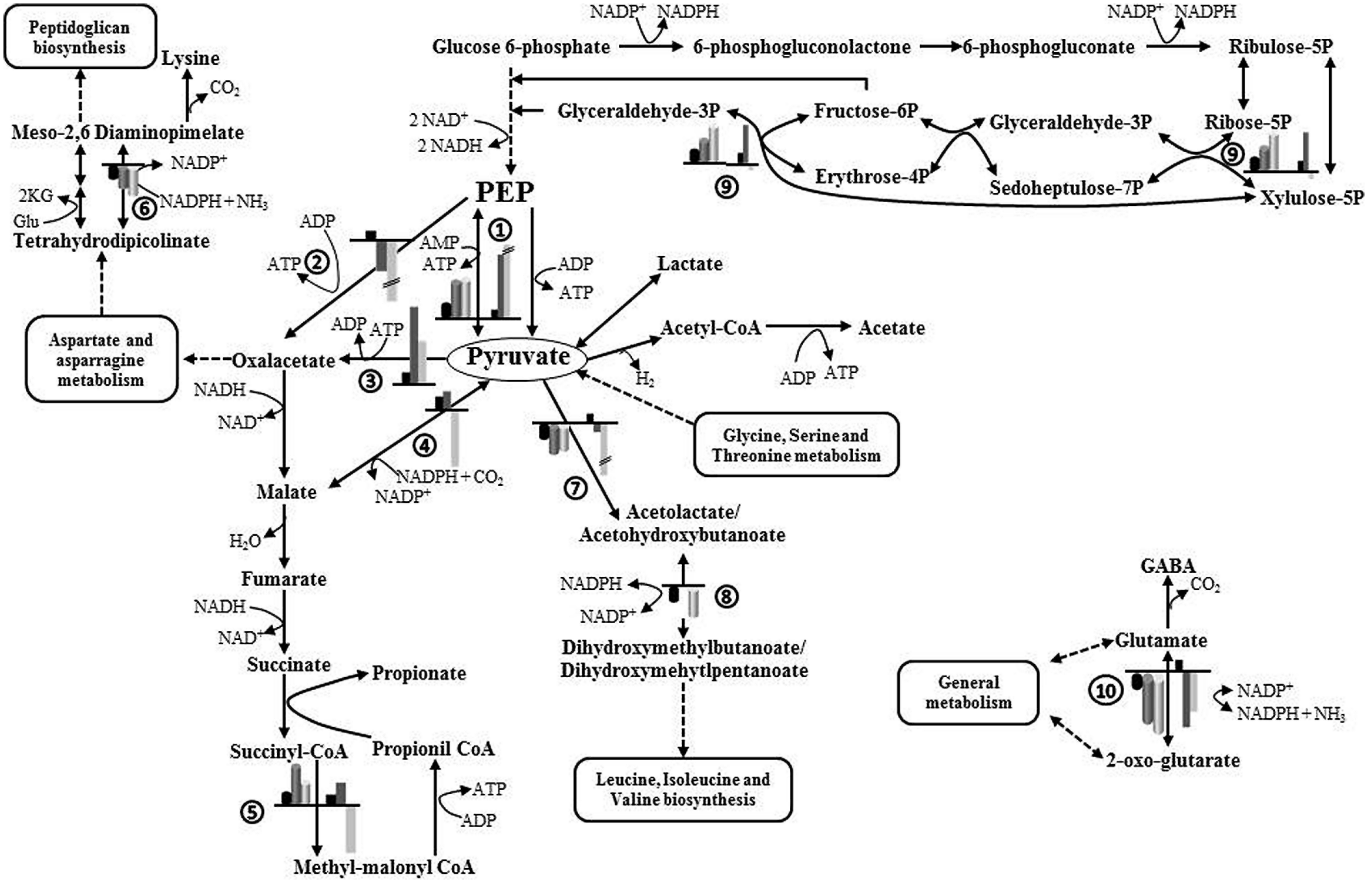
**Schematic representation of the main metabolic pathways affected by the availability of carbohydrates added to the culture medium (glucose, EPS E44, and EPS R1) during growth of *B. fragilis*.** Production ratios of enzymes catalyzing different steps in the presence of EPS with respect to glucose are indicated by cylindrical bars. Fold changes in the expression of genes in the presence of EPS with respect to glucose are indicated by rectangular bars. Black bars, glucose; dark gray bars, EPS E44; light gray bars, EPS R1. Ratios greater than sevenfold are shown with a height break. Enzymes: (1) pyruvate phosphate dikinase, (2) PEP carboxykinase, (3) pyruvate carboxylase, (4) malate dehydrogenase, (5) methyl-malonylCoA mutase, (6) diaminopimelate dehydrogenase, (7) acetolactate synthase, (8) ketol-acid reductoisomerase, (9) transketolase, (10) NADPH-dependent glutamate dehydrogenase. 2KG, 2-oxoglutarate; Glu, glutamate; PEP, phosphoenolpyruvate.

**Figure 4** depicts a schematic representation of the main affected metabolic pathways of *B. fragilis* and the proteins involved. As shown in **Table 2**, several enzymes related to the metabolism of carbohydrates were upregulated in the presence of one or two EPS: alpha-glucosidase and galactokinase-enzymes related to the release of 1→4 α-D-glucose from complex carbohydrates and with the phosphorylation of D-galactose facilitating its entry into several metabolic routes, respectively; transketolase-enzyme from the pentose phosphate pathway that catalyzes the transfer of aldehyde or ketonic groups between monosaccharides of different carbon residues; and the pyruvate phosphate dikinase-catalyzing the interconversion of phosphoenolpyruvate (PEP) and pyruvate. The overproduction of α-glucosidase was consistent with the higher enzymatic activity found in CFE of *B. fragilis* grown with EPS added (**Figure 2**).

Several proteins participating in the metabolism of different amino acids, some of which catalyze redox reactions, were underproduced in cultures of *B. fragilis* when incubated with EPS fractions: diaminopimelate dehydrogenase–oxidoreductase participating in one of the two redundant pathways leading to the formation of lysine and diaminopimelic acid; NAD(P)H-dependent glutamate dehydrogenase–oxidoreductase participating in the assimilation of ammonia to form glutamate; ketol-acid reductoisomerase and acetolactate synthase-two enzymes, oxidoreductase and transferase respectively, involved in the biosynthesis of isoleucine, valine, and leucine. Concomitantly, several proteins related to the translation and elongation steps of protein synthesis, including ribosomal proteins and elongation factors, were underproduced in the presence of one or both EPS. At the same time a translation initiation inhibitor was overproduced in the presence of EPS R1 (**Table 2**). These metabolic changes suggest a modification of the metabolism of amino acids, proteins, and peptides in *B. fragilis* when the microorganism is grown in the presence of bifidobacterial EPS fractions as compared to glucose.

Other proteins whose production was affected by carbohydrates available in the culture medium include: (i) the large subunit of the methylmalonil-CoA mutase-enzyme participating in the synthesis of propionate from succinate, the production of which was enhanced with EPS fractions, (ii) the major outer membrane protein OmpA which was overproduced only with EPS R1, and (iii) ferritin which was overproduced in the presence of both EPSs (**Table 2**). Additionally, several hypothetical proteins of unknown function in *B. fragilis* were differentially produced in the presence of one or two EPS in the culture medium. Possibly, proteins BF2494, BF2537, and BSGH_4061 according to BLAST homology with membrane proteins with TRP domain, a histidine kinase membrane protein and heat-shock protein respectively, in other *Bacteroides* species (data not shown).

### Changes in Gene Expression by RT-qPCR

The relative expression of genes coding for some proteins selected on the basis of the 2D-DIGE experiments was further analyzed by qRT-PCR (Supplementary Figure S1). The genes for the NAD(P)H-dependent glutamate dehydrogenase (*gdhB*) and the acetolactate synthase (*ilvB*) showed downregulation in the presence of both EPSs, whereas the gene coding for the major outer membrane protein OmpA (*ompA*) and the pyruvate phosphate dikinase (*ppdK*) displayed increased expression under the same conditions. These results are in agreement with the differential protein production found by proteomic analyses. The transketolase gene (*tktB*) appeared upregulated in the presence of the EPS E44 and slightly downregulated with EPS R1, which partly supports the behavior found for the corresponding protein in 2D-DIGE experiments. However, in spite of the clearly higher molar proportions of propionate obtained by us in cultures of *B. fragilis* in the presence of EPS E44 and R1 and the enhanced production of the methyl-malonyl-CoA mutase enzyme in such conditions, only a moderate overexpression and a slight underexpression of the corresponding gene (*mutB*) were respectively obtained in cultures of *B. fragilis* with EPS E44 and EPS R1 at the OD_600_ max of cultures. Attempts to analyze the expression of *mutB* at earlier points during growth proved unsuccessful due to the lack of sufficient RNA for RT-qPCR determinations (data not shown).

In addition, we assessed the expression of three genes playing a key role in the entry of PEP/pyruvate into the succinate-propionate pathway in *Bacteroides* (**Figure 4** and Supplementary Figure S1) and which did not display detectable changes in 2D-DIGE gels. One of them, the PEP carboxykinase (*pckA*) is responsible for the carboxylation of PEP to oxaloacetate with ATP formation. Pyruvate carboxylase (*pyc*) catalyzes the synthesis of oxaloacetate by carboxylation of pyruvate with ATP consumption, and malate dehydrogenase (*mdh*) catalyzes the formation of malic acid from pyruvate with consumption of reducing power. *PckA* was underexpressed in the presence of both EPS, whereas *pyc* was overexpressed and a slight overexpression or underexpression was respectively found for *mdh* with EPS E44 and R1.

### Intracellular Redox Balance

The redox ratio was significantly lower (*P* < 0.05) in cultures added with EPS E44 and R1 than in cultures in the presence of glucose, indicating a more reduced intracellular state of *B. fragilis* in the former two conditions. The NAD(P)H-associated fluorescence was significantly higher (*P* < 0.05) in cultures of *B. fragilis* ran with EPS fractions than in cultures with glucose whereas no significant differences (*P* > 0.05) were found in the fluorescence associated to FAD between cultures with EPS and glucose (**Table 3**). This indicated that the lower redox ratio found in our cultures when *B. fragilis* was grown in the presence of EPS was mainly due to an increase of the pool of intracellular NAD(P)H rather than to variations in FAD intracellular levels.

**Table 3:** Intracellular redox ratios of NAD(P)H- and FAD fluorescence associated with *B. fragilis* grown in the presence of additional glucose, EPS E44 and EPS R1.

	Glucose	EPS E44	EPS R1
Redox ratio	0.70 ± 0.03^c^	0.59 ± 0.06^b^	0.46 ± 0.09^a^
Fluorescence (NAD(P)H)	2.89 ± 0.99^a^	7.07 ± 0.93^b^	9.21 ± 0.90^c^
Fluorescence (FAD)	6.30 ± 1.71	10.38 ± 2.97	8.31 ± 3.48

*Letters indicate significant differences between cultures with different carbohydrates (*P* < 0.05). The redox ratio was calculated according to the formula: 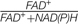.*

## Discussion

The majority of *B. fragilis* metabolic studies go back 2–3 decades ago, and in those studies the influence of external nutritional conditions were not considered. Under the culture conditions used in the present study, *B. fragilis* was able to grow in the presence of glucose and bifidobacterial EPS, the pH decrease in the culture medium being more pronounced with glucose than with the bacterial polymers. The slow growth of *B. fragilis* in the presence of EPS and the need of enough biomass and comparable cell counts for proteomic analyses, prompted us to choose late exponential phase for most of the experiments in this study (growth curves shown in Rios-Covian et al., 2013).

Molar proportions of propionic acid in cultures of *B. fragilis* in the presence of EPS or in the absence of additional carbohydrates were considerably higher than in cultures with glucose, which occurred with a concomitant reduction in the proportion of succinic, formic, and lactic acids. These results essentially confirmed our previous findings and those of other authors indicating that the ratio of propionic to succinic acid in cultures of *Bacteroides* at advanced stages of growth is higher in complex carbon sources or under carbohydrate-shortage conditions than in medium with glucose (Kotarski and Salyers, 1981; Rios-Covian et al., 2013; Adamberg et al., 2014). In addition, the higher production of SCFA and organic acids in cultures with EPS compared to cultures without carbohydrate supplementation suggests a possible utilization of these polymers as fermentable substrates (Rios-Covian et al., 2013).

The α-D-glucosidase is one of the most abundant glycoside hydrolases produced by *B. fragilis*; the enzyme, located in the periplasmic space (Berg et al., 1980), catalyzes the hydrolysis of terminal, non-reducing (1→4) linked α-D glucose from complex carbohydrates. We found that the α-glucosidase activity was virtually absent both in culture supernatants and in CFEs when grown in glucose or in the absence of additional carbohydrates, but was present in CFEs from *B. fragilis* cultured in the presence of EPSs. The EPS 44 fraction contains two polymers of different molar mass which are composed of glucose and galactose in proportion 1:1 (Salazar et al., 2009a) whereas EPS R1 is formed by three polymers of different molar mass which are composed by glucose, galactose, and rhamnose in proportions 1:1:1.5 (Ruas-Madiedo et al., 2010). Since both EPS fractions contain glucose in their composition (Salazar et al., 2009a; Ruas-Madiedo et al., 2010), α-glucosidase may be involved in the utilization of these EPS fractions by *B. fragilis*. Proteomics confirmed the overproduction of the α-glucosidase in the presence of both EPSs and also revealed that galactokinase, another enzyme related to the metabolism of carbohydrates, was overexpressed in the presence of EPS R1. The overproduction of these two enzymes suggests an expansion in the metabolic ability of *B. fragilis* to take advantage of complex carbohydrates when the microorganism is grown in the absence of readily fermentable carbohydrates. To this respect it is worth mentioning that galactose is one of the most abundant monosaccharides in intestinal mucin (Robbe et al., 2003) whereas starch (backbone of 1→4 α-D-glucose) is the main source of complex carbohydrates in the human diet.

### Amino Acids Metabolism

*Bacteroides* can produce BCFA from amino acids and proteins (Macfarlane et al., 1991; Smith and Macfarlane, 1998) and recent data showed that *B. fragilis* can release them in the presence of EPS (Rios-Covian et al., 2013). Remarkably, isovaleric and isobutyric acids, originating from the catabolism of leucine and valine respectively, were formed in *B. fragilis* cultures with EPS but not glucose. Our experimental results also point to an enhanced consumption of the pool of amino acids present in the culture medium and a higher release of ammonia in the presence of EPS as compared to glucose. The high removal of asparagine from supernatants and the absence of residual asparagine and alanine in CFEs indicated the efficient conversion of asparagine to aspartic acid rendering alanine that is finally transformed to pyruvic acid, under all culture conditions tested. An enhanced removal of threonine from the culture medium also occurred in the presence of EPS, whereas glycine accumulated at lower levels than in the presence of glucose. Glycine and serine are intermediate in the degradation of threonine toward their incorporation at the level of pyruvate; therefore these three amino acids may serve as a source of pyruvate for *B. fragilis* grown in the presence of EPS. Moreover, the enzymes ketol-acid reductoisomerase and acetolactate synthase, that participate in the biosynthetic pathway of isoleucine, leucine (amino acids removed from the culture medium by *B. fragilis* under all conditions) and valine from pyruvate were found to be underproduced in EPS as compared to glucose. These facts suggest that in *B. fragilis* the metabolism of some amino acids rendering pyruvate, a key intermediate of central catabolism, was activated whereas the synthesis of those using pyruvate as a precursor may be partly inhibited in the presence of bacterial EPS. Interestingly, threonine is a major component of the human intestinal mucin, together with asparagine and serine (Aksoy and Akinci, 2004). This result points out an adaptation of *B. fragilis* metabolism for improving utilization of intestinal mucin, as previously reported by Adamberg et al. (2014) for *Bacteroides thetaiotaomicron* subjected to amino acid starvation.

A clear underproduction of the enzyme NAD(P)H-dependent glutamate dehydrogenase was found in the presence of EPS. *B. fragilis* possesses two distinct glutamate dehydrogenases that play a fundamental role in nitrogen assimilation (Yamamoto et al., 1987; Baggio and Morrison, 1996). The NAD(P)H-dependent glutamate dehydrogenase catalyzes the assimilation of ammonia by reductive amination of α-ketoglutarate to form L-glutamate, whereas the NADH-dependent glutamate dehydrogenase catalyzes the reverse reaction. The NADH-dependent enzyme displays a basal activity that increases at high organic nitrogen concentration whereas in such conditions the NAD(P)H-dependent enzyme was inhibited (Abrahams and Abratt, 1998). In addition, glutamic acid is a metabolic intermediate in the synthesis of different amino acids and other compounds, its concentration in CFE being the highest found by us under all conditions assayed. It is then plausible that the catabolic reaction in the direction of ammonia and α-ketoglutarate formation from glutamate would predominate in cultures with EPS, as compared to cultures performed with glucose. The α-ketoglutarate formed could then serve as a substrate for transamination reactions in *B. fragilis* (Abrahams and Abratt, 1998). On the other hand, GABA was present in significantly lower levels in the culture supernatants of *B. fragilis* incubated with additional EPS, as compared with the cultures in the presence of glucose. GABA is formed from glutamate in a single reaction step mediated by a glutamate decarboxylase enzyme. An acid resistance mechanism has been described in some intestinal bacteria in which a molecule of extracellular glutamate is antiported with an intracellular proton and converted to GABA that is subsequently exchanged for another extracellular glutamate (Cotter et al., 2001). Likely, under mild acidic conditions occurring in cultures with EPS as compared to glucose, the glutamate decarboxylase pathway is not being used by *B. fragilis*, thus GABA is not formed.

Aspartic acid is a precursor in the biosynthetic pathway of lysine and meso-diaminopimelic acid. Lysine takes part of the pentapeptide bridge of the peptidoglycan in Gram-positive bacteria, and is replaced by meso-diaminopimelic acid in Gram negatives. *B. fragilis* has two redundant biosynthetic pathways for the synthesis of meso 2,6-diaminopimelate: one is catalized by the oxidorreductase diaminopimelate dehydrogenase with consumption of ammonia and NADPH and the other occurs in two steps, catalyzed by an aminotransferase and an epimerase, respectively. The diaminopimelate dehydrogenase was found underproduced by proteomic analyses when *B. fragilis* was grown in the presence of EPS, thus favoring in such conditions the alternative route which generates a molecule of α-ketoglutarate from glutamate to form 2,6-diaminopimelate (Hudson et al., 2011). The α-ketoglutarate could then serve as substrate for cellular transamination reactions, as commented before. Our results also support the idea that the amino acids are more efficiently removed by *B. fragilis* from the culture medium under shortage conditions of readily fermentable carbohydrates, as would be the case when this microorganism is incubated with EPS. In this respect, the strong influence that available carbohydrates exert on peptide and amino acids metabolism in *Bacteroides* and other intestinal bacteria is known (Macfarlane et al., 1991; Smith and Macfarlane, 1998; Adamberg et al., 2014).

Finally, some proteins involved in translation, ribosomal structure, and biogenesis were underproduced whereas a translation initiation inhibitor was upregulated in *B. fragilis* in the presence of EPS as compared to glucose, indicating a slowdown of the protein synthesis in this bacterium. Under these conditions the intracellular pool of amino acids can be maintained by transamination reactions involving α-ketoacids and amino acids collected from the culture medium.

### Energetics, Metabolism, and Redox Balance

Using proteomics we could not find variations in the production of glycolytic enzymes. However, overproduction of pyruvate phosphate dikinase was confirmed by proteomics and gene expression analyses. This enzyme catalyzes the interconversion of PEP and pyruvate, with ATP formation in the forward direction. Although pyruvate phosphate dikinase functions in the gluconeogenesis direction in several organisms, the pyruvate formation seems to be favored at moderate acidic conditions in *Bacteroides symbiosus* (optimum pH in the forward direction 6.6 and 7.2-7.8 in the reverse direction; Reeves, 1971), as may also occur in some parts of the human colon ecosystem (Vertzoni et al., 2010). The dominant route of microbial propionate synthesis in the gut is the succinate–propionate pathway via formation of methyl-malonyl-CoA, which is present in *Bacteroides* (Reichardt et al., 2014). In our case, it seems that when *B. fragilis* is growing in glucose, a significant part of PEP would be converted to oxaloacetate, malate, and pyruvate being then formed by decarboxylation of oxaloacetate (Macy et al., 1978). However, we found that the gene coding for PEP carboxykinase (catalyzing carboxylation of PEP to oxaloacetate) was under-expressed when *B. fragilis* was grown in EPS as compared to glucose; in such conditions the pyruvate carboxylase (catalyzing carboxylation of pyruvate to oxaloacetate) was over-expressed and the malate dehydrogenase displayed slight variation in its expression in cultures with EPS when compared to cultures with glucose. Taking into account that an increase in the degradation of amino acids leading to pyruvate and a partial inhibition of the synthesis of amino acids from pyruvate seems to occur in *B. fragilis* cultured in the presence of EPS, a relative accumulation of pyruvate and a relative shortage of PEP originating from carbohydrates may happen under such conditions. Therefore, it is plausible that in the presence of EPS a significant part of oxaloacetate would be formed from pyruvate. The pentose-phosphate pathway generates pentoses and reducing power in the form of NADPH. In the presence of glucose, the succinate-propionate pathway keeps the intracellular redox balance of NAD^+^/NADH + H^+^ through the reoxidation of two moles of NADH generated in the conversion of glucose to PEP in glycolysis. However, under conditions of carbohydrate shortage and enhanced amino acid removal from the medium, as we propose occurs when *B. fragilis* grows in a BM with additional EPS, a redox imbalance may happen due to insufficient reducing power supply from glycolysis. Under such conditions, the main input for intermediates of the succinate–propionate pathway would be the amino acids incorporated at the level of pyruvate and therefore a relative discompensation between NADH/NADPH may occur. The flux enhancement through the pentose-phosphate pathway leads to NADPH synthesis and overexpression of transketolase in cultures with EPS, providing the additional reducing power needed to maintain the cellular redox balance while accomplishing essential metabolic functions. The lower redox ratio associated with higher levels of NADH that we found in cells of *B. fragilis* grown with EPS, agrees with this hypothesis. Moreover, a metabolic redirection of NADH to NADPH has been shown in *Pseudomonas fluorescens* under oxidative stress (Singh et al., 2008). The metabolic ability to inter-convert both nicotinamide dinucleotides, although not yet demonstrated in *Bacteroides*, may ensure keeping the intracellular NADH and NADPH pools as an adaptation to external stimuli.

### The Unknown Balance between Beneficial and Detrimental Effects in the Intestinal Ecosystem

A ferritin was overproduced in *B. fragilis* grown in the presence of EPS as compared to glucose. Ferritin-like proteins sequestrate iron in response to the presence of oxygen, playing an important role in the pathogenicity of this bacterium (Rocha and Smith, 2013). On the other hand, the major outer membrane protein OmpA was overproduced in cultures of *B. fragilis* grown with EPS R1. In other microorganisms, OmpA functions as a porin that enables the passage of nutrients and different compounds into the cell (Reeves et al., 1996, 1997). OmpA has also been implicated in cell functions related with pathogenicity (Soulas et al., 2000; Smith et al., 2007). In *B. fragilis*, the involvement of OmpA1 in maintaining cell structure as well as in the release of cytokines by murine splenocytes has been demonstrated (Magalashvili et al., 2008; Wexler et al., 2009). Whether OmpA overproduction is related with an improved ability of *B. fragilis* to obtain nutrients from the environment, and whether this is related with beneficial or detrimental effects for the host is, at present, unknown.

## Conclusion

The results presented provide an insight into the physiological and molecular mechanisms that allow *B. fragilis* to adapt to an environment where EPSs are the main source of carbohydrates. Protein expression with different functions was modulated, and the intracellular and extracellular pattern of given amino acids, redox balance, and α-glucosidase activity were affected by the external environment. Three main events, namely the activation of amino acid catabolism, enhancement of the transketolase reaction from the pentose-phosphate pathway and the activation of the succinate–propionate pathway for propionic formation, suggest a metabolic shift in this bacterium toward the generation of more reducing power, and to optimize the ATP yield. These changes would represent a metabolic adaptation of *B. fragilis* to take advantage of carbohydrates and proteins available in the intestinal environment, such as mucin. Recent results from our group indicated that in the presence of EPS, propionic acid production increased in human fecal cultures (Salazar et al., 2008), whereas the growth of bifidobacteria was enhanced by *B. fragilis* (Rios-Covian et al., 2013). Both effects are generally considered as health promoters in the human gut, but other potentially harmful effects from the couple EPS/*B. fragilis* cannot be ruled out and deserve further investigation. Our work has revealed a number of intriguing topics for future research regarding the relationship of probiotics and prebiotics with the symbiont or mutualistic microbial populations of the human intestinal ecosystem.

## Conflict of Interest Statement

The authors declare that the research was conducted in the absence of any commercial or financial relationships that could be construed as a potential conflict of interest.
