# Supplementary material for: Different metabolic features of Bacteroides fragilis growing in the presence of glucose and exopolysaccharides of bifidobacteria
Source: Front Microbiol. 2015 Aug 18;6:825. doi: 10.3389/fmicb.2015.00825 (PMC4539542; doi:10.3389/fmicb.2015.00825)
Supplement: Supplementary file 1 [file Table_1.DOCX]

**SUPPLEMENTARY MATERIAL TABLE S1**

**Tittle:** Different metabolic features of *Bacteroides fragilis* growing in the presence of glucose and exopolisaccharides of bifidobacteria as fermentable carbohydrates

**Authors:** David Ríos-Covián, Borja Sánchez #, Nuria Salazar, Noelia Martínez, Begoña Redruello, Miguel Gueimonde and Clara G. de los Reyes-Gavilán *

*Address correspondence to: Clara G. de los Reyes-Gavilán, greyes_gavilan@ipla.csic.es

**Table S1**. Targeted genes and primers used for RT-qPCR in this study. Annealing temperature (Tm) was 60ºC.

| Target gene | Primer name | Primer Sequence 5’-3’ |
| --- | --- | --- |
| Glutamate dehydrogenase  (*gdhB*) | BFgdhF | GTGATGCGGAAATCATGCGT |
|  | BFgdhR | TGACGCCACAATTCAAGCA |
| Phosphoenol pyruvate kinase  (*pck*) | BFpepkF | ATCACTGAACCGACTCCGACA |
|  | BFpepR | GCAATGATAAGAATGCAGCACC |
| Acetolactate synthetase  *(ilvB)* | BFilvF | CGTGCATTCCCAGCATACC |
|  | BFilvR | TTGGGACTGTCGGCTTTGC |
| Methylmalonil-CoA mutase  (*mutB*) | BFmmmF | CCCTTACCGGTTTTCACACATT |
|  | BFmmmR | TCAGGCCGAAGTGGACAAG |
| Membrane protein OmpA  (*ompA*) | BFOmpAF | CGGTACGCGGAGCAATGT |
|  | BFOmpAR | GTGGCAGAAGTTGCCGAACA |
| Pyruvate phosphate dikinase  (*ppdK*) | BFpyrKF | GCACGTTCCTCTTCGCTCA |
|  | BFpyrKR | TAAGATCGGTTCCCAGCGTT |
| Transketolase  (*tktB*) | BFtktF | GGATCAGTGCTGTAGGAGTCGA |
|  | BFtktR | TGGAAGAGACAACCATTGCG |
| Pyruvate carboxylase  (*pyc*) | BFpyCF | CGGAGTCTCTTGCGCATTCT |
|  | BFpyCR | AAGAGGTACTTTCGCCGCTT |
| Malate dehydrogenase  (*mdh*) | BFmdhF | TGACCCGCCAACTCTACGA |
|  | BFmdhR | CTTCGGCAAATACCACAC |
