# Supplementary material for: Different metabolic features of Bacteroides fragilis growing in the presence of glucose and exopolysaccharides of bifidobacteria
Source: Front Microbiol. 2015 Aug 18;6:825. doi: 10.3389/fmicb.2015.00825 (PMC4539542; doi:10.3389/fmicb.2015.00825)
Supplement: Supplementary file 2 [file Table_2.DOCX]

**SUPPLEMENTARY MATERIAL TABLE S2**

**Tittle:** Different metabolic features of *Bacteroides fragilis* growing in the presence of glucose and exopolisaccharides of bifidobacteria as fermentable carbohydrates

**Authors:** David Ríos-Covián, Borja Sánchez, Nuria Salazar, Noelia Martínez, Begoña Redruello, Miguel Gueimonde and Clara G. de los Reyes-Gavilán *

*Address correspondence to: Clara G. de los Reyes-Gavilán, greyes_gavilan@ipla.csic.es

**Table S2.** Changes in aminoacid profile during incubation of *B. fragilis* with glucose (G), EPS (E44 and R1) and without carbohydrate source added to the culture medium (WCS). Arrows up and down indicate increase or decrease, respectively, of amino acid concentration as regards to the initial levels, during incubation. Different letters indicate significant differences among cultures in different carbohydrate sources (*P* < 0.05).

| Amino acid | Carbohydrate source | Supernatant  mM % consumption | | Cell-free extracts  (mmol / g protein ) |
| --- | --- | --- | --- | --- |
| Aspartic | G  E44  R1  WCS | -0.76±0.08^a^  -0.56±0.07^b^  -0.44±0.06^b^  0.12±0.02^c^ | -52.33±12.65↓^a^  -38.09±4.90↓^ab^  -32.68±4.93↓^b^  9.00±1.78↑^c^ | 11,83±5,31^a^  35.84±26.45^a^  37.03±11.98^a^  91.27±11.82^b^ |
| Glutamic | G  E44  R1  WCS | 0.14±0.11^b^  0.27±0.05^b^  -0.09±0.02^a^  -0.09±0.04^a^ | 3.49±3.23↑^b^  6.15±1.67↑^b^  -2.17±0.52↓^a^  -2.15±1.01↓^a^ | 139.11±42.62^a^  166.06±31.72^a^  184.27±38.88^a^  285.30±15.12^b^ |
| Asparagine | G  E44  R1  WCS | -1.04±0.14  -0.99±0.16  -1.02±0.21  -0.94±0.00 | -100.00±0.00↓  -100.00±0.00↓  -100.00±0.00↓  -100.00±0.00↓ | 0.00±0.00  0.00±0.00  0.00±0.00  0.00±0.00 |
| Serine | G  E44  R1  WCS | -0.28±0.44^b^  -1.05±0.12^a^  -0.63±0.25^ab^  -0.20±0.05^b^ | -11.28±-19.64↓^b^  -54.68±18.66↓^a^  -33.41±16.58↓^ab^  -10.50±2.75↓^b^ | 7.42±9.66  12.28±21.27  0.80±1.60  9.81±11.45 |
| Histidine | G  E44  R1  WCS | 0.11±0.01^a^  0.21±0.03^b^  0.18±0.05^ab^  0.14±0.01^ab^ | 31.72±8.60↑^a^  89.98±15.29↑^c^  75.61±6.32↑^bc^  59.49±4.44↑^b^ | 0.60±1.19  0.90±1.56  1.69±1.96  0.00±0.00 |
| Glycine | G  E44  R1  WCS | 1.21±0.34^b^  0.51±0.10^a^  0.40±0.04^a^  0.38±0.04^a^ | 84.07±33.00↑^b^  34.60±7.04↑^a^  28.83±2.11↑^a^  27.81±2.98↑^a^ | 90.24±16.82  92.03±24.68  97.91±23.37  114.48±11.11 |
| Threonine | G  E44  R1  WCS | 1.64±0.07^c^  -0.33±0.15^a^  -0.27±0.06^a^  0.22±0.04^b^ | 133.27±10.36↑^c^  -27.62±18.20↓^a^  -22.35±5.41↓^a^  17.58±2.88↑^b^ | 15.78±13.67  2.10±3.63  5.39±4.97  0.00±0.00 |
| Arginine | G  E44  R1  WCS | 0.27±0.22  0.54±0.14  0.40±0.06  0.41±0.03 | 28.58±20.48↑^a^  65.33±7.60↑^b^  50.38±15.68↑^ab^  54.09±4.25↑^ab^ | 1.78±2.28^a^  28.22±4.33^ab^  29.11±11.28^b^  12.15±11.31^ab^ |
| GABA | G  E44  R1  WCS | 0.52±0.22^b^  0.02±0.01^a^  -0.05±0.11^a^  0.08±0.00^a^ | 521.51±283.81↑^b^  16.88±14.39↑^a^  14.11±15.40↑^a^  81.61±1.99↑^a^ | 66.04±33.62^b^  0.00±0.00^a^  0.49±0.57^a^  0.00±0.00^a^ |
| Alanine | G  E44  R1  WCS | 1.35±0.45  0.56±0.30  0.58±1.02  0.27±0.14 | 28.43±13.36↑  10.80±6.50↑  8.50±13.80↑  6.03±3.15↑ | 0.00±0.00  0.00±0.00  0.00±0.00  0.00±0.00 |
| Proline | G  E44  R1  WCS | 1.38±1.23  0.99±0.44  0.17±0.39  0.16±0.10 | 50.43±50.30↑  20.38±8.43↑  3.41±8.17↑  3.60±2.31↑ | 109.99±41.22^a^  251.05±14.75^b^  238.07±48.66^b^  366.59±19.34^b^ |
| Tyrosine | G  E44  R1  WCS | 0.23±0.15^b^  0.01±0.08^ab^  0.01±0.10^ab^  -0.03±0.03^a^ | 35.16±25.85↑^b^  0.83±10.43↑^a^  0.90±11.80↑^a^  -3.62±3.39↓^a^ | 122.93±12.22^a^  99.77±14.73^a^  112.96±25.47^a^  220.81±17.88^b^ |
| Valine | G  E44  R1  WCS | 0.15±0.09  0.07±0.15  -0.03±0.03  0.06±0.04 | 8.66±6.03↑  3.28±9.86↑  -1.77±1.18↓  3.74±2.38↓ | 101.67±5.64^a^  80.95±34.37^a^  105.64±19.56^a^  185.28±2.78^b^ |
| Methionine | G  E44  R1  WCS | -0.05±0.01  0.01±0.06  -0.03±0.00  -0.01±0.00 | -12.65±3.72↓  -0.02±15.06↓  -5.49±3.84↓  -1.42±0.67↓ | 1.77±0.81  7.20±0.02  7.53±1.33  6.82±3.01 |
| Tryptophan | G  E44  R1  WCS | -0.01±0.01^a^  0.03±0.02^b^  0.02±0.01^b^  0.01±0.01^ab^ | -3.87±6.84↓^a^  15.15±11.19↑^b^  12.57±6.14↑^b^  4.92±3.28↑^ab^ | 0.00±0.00  0.00±0.00  0.22±0.44  0.00±0.00 |
| Isoleucine | G  E44  R1  WCS | -0.22±0.16  -0.25±0.19  -0.27±0.04  -0.11±0.03 | -12.18±7.88↓  -16.26±15.49↓  -16.36±2.91↓  -7.08±2.25↓ | 58.51±2.38^a^  43.69±28.92^a^  63.36±12.58^a^  110.71±6.83^b^ |
| Leucine | G  E44  R1  WCS | -0.71±0.27  -0.67±0.39  -0.73±0.19  -0.29±0.07 | -21.61±5.87↓  -22.70±17.28↓  -22.81±2.70↓  -10.06±2.39↓ | 32.32±13.33  30.79±32.46  50.97±19.89  77.70±25.53 |
| Phenylalanine | G  E44  R1  WCS | -0.14±0.05  -0.10±0.07  -0.17±0.09  -0.06±0.03 | -9.81±3.66↓  -7.99±7.04↓  -11.58±3.22↓  -4.97±2.53↓ | 25.24±4.77  23.21±20.13  36.06±10.15  49.83±9.39 |
| Ornithine | G  E44  R1  WCS | 0.20±0.13^c^  0.01±0.02^b^  0.08±0.03^bc^  -0.37±0.01^a^ | 226.93±159.69↑^b^  7.60±17.85↑^a^  65.14±26.49↑^a^  -60.43±1.35↓^a^ | 14.45±11.61^b^  0.00±0.00^a^  11,99±3,18^ab^  0.00±0.00^a^ |
| Lysine | G  E44  R1  WCS | 0.39±0.10^a^  1.01±0.25^b^  0.67±0.26^ab^  0.82±0.05^b^ | 34.94±10.18↑^a^  79.89±8.65↑^b^  57.00±27.36↑^ab^  72.87±4.65↑^b^ | 39.96±7.33^a^  85.79±26.98^ab^  105.53±21.72^b^  132.88±10.83^c^ |
