# Supplementary material for: Different metabolic features of Bacteroides fragilis growing in the presence of glucose and exopolysaccharides of bifidobacteria
Source: Front Microbiol. 2015 Aug 18;6:825. doi: 10.3389/fmicb.2015.00825 (PMC4539542; doi:10.3389/fmicb.2015.00825)
Supplement: Supplementary file 3 [file Figure1.PDF]

SUPPLEMENTARY MATERIAL Figure S1

Tittle: Different metabolic features of *Bacteroides fragilis* growing in the presence of glucose and exopolisaccharides of bifidobacteria as fermentable carbohydrates

Authors: David Ríos-Covián, Borja Sánchez, Nuria Salazar, Noelia Martínez, Begoña Redruello, Miguel Gueimonde and Clara G. de los Reyes-Gavilán *

*Address correspondence to: Clara G. de los Reyes-Gavilán,

greyes_gavilan@ipla.csic.es

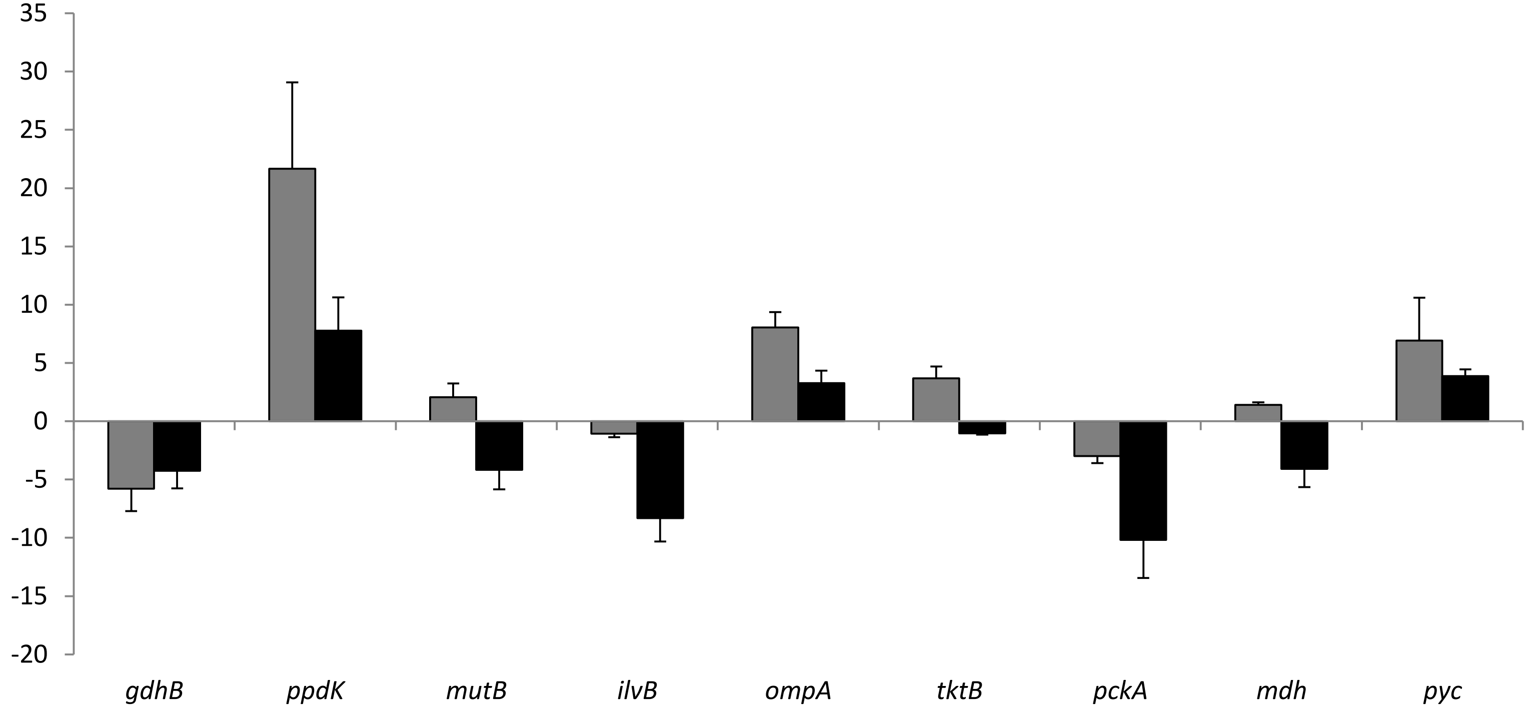

Figure S1. Relative expression levels of different genes in cultures of *B. fragilis* grown in the presence of bifidobacterial EPS fractions with respect to the cultures in glucose. Glutamate dehydrogenase (*gdhB)*, pyruvate phosphate dikinase (*ppdK)*, methyl-malonyl CoA mutase (*mutB*)*,* acetolactate synthetase (*ilvB)*,membrane protein OmpA (*ompA)*, transketolase (*tktB*), phosphoenolpyruvate carboxykinase (pckA), malate dehydrogenase (*mdh*) and pyruvate carboxylase (*pyc*) genes. Grey bars, changes in gene expression levels in the presence of EPS E44 with respect to glucose. Black bars, changes in gene expression levels in the presence of EPS R1 with respect to glucose.
